# An extreme internal solitary wave event observed in the northern South China Sea

**DOI:** 10.1038/srep30041

**Published:** 2016-07-21

**Authors:** Xiaodong Huang, Zhaohui Chen, Wei Zhao, Zhiwei Zhang, Chun Zhou, Qingxuan Yang, Jiwei Tian

**Affiliations:** 1 Physical Oceanography Laboratory/CIMST, Ocean University of China and Qingdao National Laboratory for Marine Science and Technology, Qingdao, China

## Abstract

With characteristics of large amplitude and strong current, internal solitary wave (ISW) is a major hazard to marine engineering and submarine navigation; it also has significant impacts on marine ecosystems and fishery activity. Among the world oceans, ISWs are particular active in the northern South China Sea (SCS). In this spirit, the SCS Internal Wave Experiment has been conducted since March 2010 using subsurface mooring array. Here, we report an extreme ISW captured on 4 December 2013 with a maximum amplitude of 240 m and a peak westward current velocity of 2.55 m/s. To the authors’ best knowledge, this is the strongest ISW of the world oceans on record. Full-depth measurements also revealed notable impacts of the extreme ISW on deep-ocean currents and thermal structures. Concurrent mooring measurements near Batan Island showed that the powerful semidiurnal internal tide generation in the Luzon Strait was likely responsible for the occurrence of the extreme ISW event. Based on the HYCOM data-assimilation product, we speculate that the strong stratification around Batan Island related to the strengthening Kuroshio may have contributed to the formation of the extreme ISW.

Internal solitary waves (ISWs), characterized by meso-fine scale, large amplitude and strong current velocity, are ubiquitous features in stratified oceans^1^,^2^. Like tornadoes in the atmosphere, ISWs provoke remarkable changes in ocean current and thermal structures in a relatively short time (tens of minutes). Therefore, ISW is a major environmental disaster threatening the safe operation of offshore oil rigs^3^ and underwater vehicles. Bringing the nutrients and plankton near the sea bottom into the upper layer, ISWs are also important to marine ecosystems^4^,^5^ and fishery activity^6^ in the ocean. Moreover, the vertical shear of the horizontal current related to ISW is often energetic, which can produce instability and result in enhanced vertical mixing^7^,^8^.

Knowledge of ISWs, especially extreme ISWs, is important for assessing their impacts on offshore oil rigs and underwater vehicles, as well as understanding their roles in the variations of marine ecosystems and fishery activity. ISWs are active in marginal seas, particularly in the northern South China Sea (SCS), the Sulu Sea, the Andaman Sea, the Washington Shelf, the North West Shelf of Australia, the Strait of Gibraltar, and Massachusetts Bay^3^,^9^,^10^,^11^,^12^,^13^,^14^. Field investigations showed that ISWs in these regions often displayed amplitudes ranging from tens to one hundred of meters. To the authors’ best knowledge, the strongest ISW among the world oceans ever reported so far was captured in the northern SCS^15^. That particular ISW had a peak horizontal velocity of 2 m/s and was estimated to have an amplitude of 170 m; it carried kinetic energy (KE) and available potential energy (APE) of 2.2 and 1.48 GJ/m, respectively^15^.

With large amplitudes and strong current velocities, ISWs in the northern SCS have been a hotspot for oceanic wave studies over the past decades^16^,^17^,^18^. By using subsurface mooring array equipped with current velocity and temperature instruments, we have been conducting the SCS Internal Wave Experiment (SIWE) from March 2010 onward, which covers a broad region with frequent ISW activities (116.2–121.9°E, 20.2–21.7°N). During the experiment period between March 2010 and June 2014, a total of 4771 ISW episodes were observed, which is the largest collection of the ISW dataset in the SCS. In this paper, the strongest ISW therein with an amplitude of 240 m and a peak westward current velocity of 2.55 m/s is reported.

## Results

### Characteristics of the extreme ISW event

**Figure 1:**
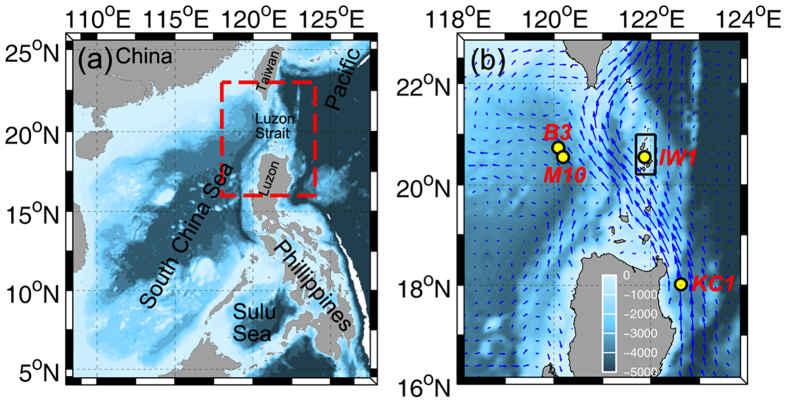
Bathymetry around the Survey region. **(a)** Topography of the SCS and the western Pacific Ocean. The red dashed box is enlarged in (**b**). **(b)** Topography around the Luzon Strait, where the locations of moorings IW1, M10, B3, and KC1 are indicated by yellow dots. The blue arrows denote the temporally averaged surface currents from 2010 to 2014 derived from the HYCOM product (http://hycom.org/). Bathymetry data are downloaded from https://www.ngdc.noaa.gov/mgg/global/. Figures are plotted using MATLAB R2013a (http://www.mathworks.com/) with M_Map (a mapping package, http://www.eos.ubc.ca/~rich/map.html).

**Figure 2:**
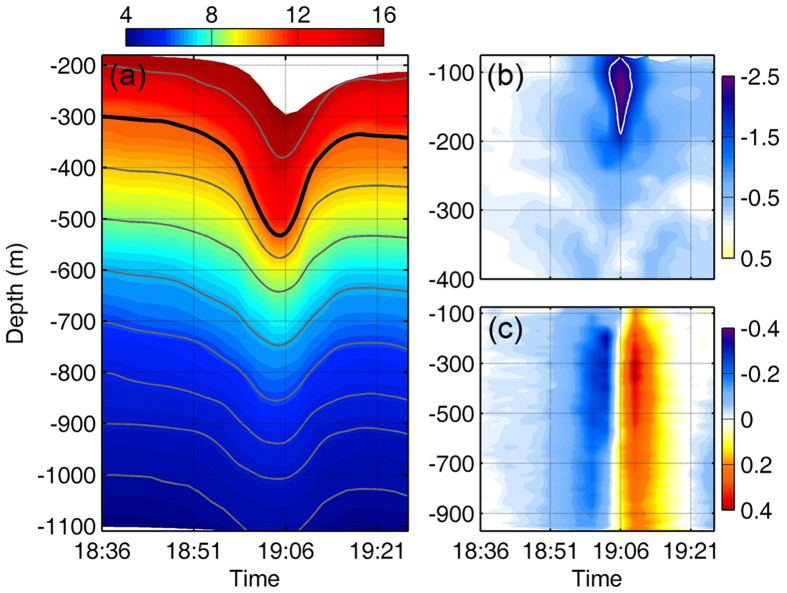
The extreme ISW event captured at M10 on 4 December 2013. **(a)** Shadings indicate the temperatures (^o^C) measured by the thermistor chains, and the lines represent the isothermal displacements at every 100 m between 200 and 1000 m. **(b)** Shadings indicate the zonal velocity anomalies (m/s) measured by the upward-looking ADCP, and the white line shows the shape of velocity anomalies with magnitude exceeding 2 m/s. **(c)** Shadings indicate vertical velocity (m/s) measured by the upward and downward-looking ADCPs. Figures are plotted using MATLAB R2013a (http://www.mathworks.com/).

On 4 December 2013, an extreme ISW event was captured by the mooring M10 (120.22°E, 20.57°N) deployed in the northern SCS at the bottom depth of 3847 m (Fig. 1). Heavy instrumentations, including two 75-kHz Acoustic Doppler Current Profilers (ADCPs), five recording current meters (RCMs), five conductivity-temperature-depth (CTD) recorders, and dozens of temperature loggers, were mounted on the mooring M10 (see Methods). Temperature observations showed that surface-layer water with high temperature sank into the lower layer during the ISW’s passage (Fig. 2a), indicating a wave of depression. At the depth of 300 m, the temperature exhibited a maximum increase of over 6 °C at the wave trough. The temperature increase exceeded 0.5 °C even at 1000 m. From isotherm contours, we can infer that the water particle at 300 m prior to the wave arrival was displaced downward to 540 m by the wave trough within 15 min, suggesting an amplitude of 240 m for the ISW. At the depth of 900 m, the downward displacement of isotherm interface reached 110 m. Between depths of 200 and 1000 m, the mean displacement of isotherm interfaces was up to 173 m. During the period between March 2010 and June 2014, 4771 ISWs have been observed by the SIWE in the northern SCS. These observed ISWs have a mean amplitude of 67 m at the depth of 300 m. Amplitude of the extreme ISW reported here is 3.6 times the 4-year-mean value.

With respect to horizontal current velocity of the extreme ISW, there existed a notably enhanced westward current zone, with a light-bulb shape around the wave trough (Fig. 2b). A peak westward current velocity of 2.55 m/s was found at 130 m at the wave trough. The strong westward current anomalies with magnitudes larger than 2 m/s extended to 190 m. At 130 m, the westward current anomalies that exceeded 2 and 1 m/s lasted for at least 5 and 12 minutes, respectively. The 4771 ISWs observed during the SIWE have a mean maximum current velocity of 0.64 m/s towards the west. So, the maximum current velocity of the extreme ISW is four times as large as the mean. Vertically, strong downward currents with a peak of 0.35 m/s were observed at the leading edge of the wave, following which the upward currents with comparable velocity magnitudes were observed at its trailing edge (Fig. 2c). Strong vertical velocities with magnitude exceeding 0.2 m/s can be found over a broad depth range between 100 and 900 m.

**Figure 3:**
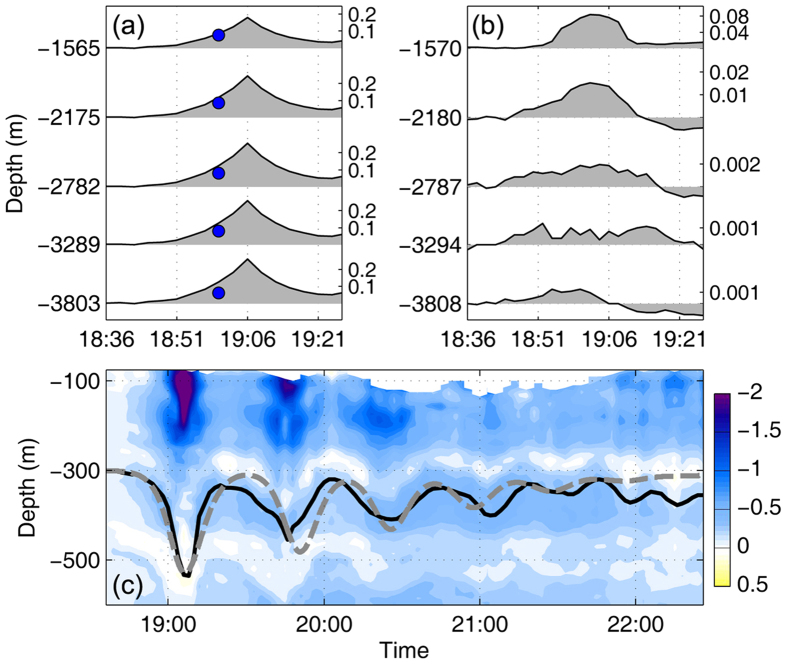
Measurements of the extreme ISW in the deep ocean and its trailing waves. **(a)** Zonal velocity anomalies (m/s) during the passage of the extreme ISW at depths of 1565, 2175, 2782, 3289, and 3803 m, respectively. The blue dots denote the zonal velocity anomalies recorded by the RCMs 6 min prior to the wave trough arrival, and the shaded areas are the zonal velocity anomalies estimated from the mode analysis method. **(b)** Temperature fluctuations (^o^C) measured by the CTDs at depths of 1570, 2180, 2787, 3294, and 3808 m, respectively. **(c)** Measurements of the trailing waves following the extreme ISW. Shadings indicate the zonal velocity anomalies (m/s) in the upper 600 m, and the bold line denotes the isotherm displacement at 300 m. The dashed line is the analytical waveform calculated by the dnoidal solution to the KdV equation. Figures are plotted using MATLAB R2013a (http://www.mathworks.com/).

In the deep water between 1565 and 3803 m, notable eastward currents with magnitude of 6–8 cm/s (blue dots in Fig. 3a) were observed 6 min prior to the wave trough arrival by the RCMs working at a burst mode. The zonal current velocity in the lower layer showed opposite direction to that in the upper layer, corresponding to the mode-1 baroclinic structure. With sampling time interval of 30 min, the measurements of the RCMs missed the peak current velocity of the ISW at the wave trough. Here, we estimate the ISW current velocity from 1565 to 3803 m at the wave trough using the mode analysis method (see Methods). As shown in Fig. 3a (gray shadings), the estimated maximum eastward current velocity associated with the extreme ISW could reach 0.2 m/s. In the meantime, the deep-water CTD measurements with sampling interval of 2 min revealed significant temperature increases (Fig. 3b) during the passage of the extreme ISW at the depths of 1570, 2180, 2787, 3294, and 3808 m, respectively. The maximum temperature increases at these depths corresponded to downward isotherm displacements of 77, 57, 35, 23, and 19 m, respectively, on the basis of temperature gradient that was calculated using the winter data of the World Ocean Atlas (WOA). Based on the above results, we suggest here that the extreme ISW exerted great influences not only on the upper ocean but also on the deep ocean.

Following the strongest ISW, six trailing waves were observed; each was weaker than its predecessor (Fig. 3c). Previous studies reported that such multi-wave ISW packets generally occurred west of 118°E around the continental shelf of the northern SCS^19^,^20^,^21^. The extreme ISW reported here was captured east of 120°E, quite close to the originating site of internal waves in the Luzon Strait (LS). Amplitudes of the 2^nd^, 3^rd^ and 4^th^ waves in the ISW packet still reached 120, 90 and 60 m, respectively. This is, therefore, another indicator for the intensity of the ISW.

In order to characterize the multi-wave ISW packet in this case, the dnoidal solution^22^,^23^ to the Kortevrieg–de Vries (KdV) equation is adopted (see Methods). The calculation shows that newborn trailing waves continuously arise from the packet tail during the evolution, and the analytical packet waveform (dashed line in Fig. 3c) most closely matches the observations when the evolution time *t* approaches 6.6 h. Toward the packet rear, time intervals among successive waves exhibit a decreasing trend due to weakened nonlinearity. Moreover, compared to the ISW packets with smaller leading waves, the dnoidal solution reveals that the extreme multi-wave ISW packet exhibits a much more compact wave pattern (see Supplementary Fig. S1). According to the KdV theory, the strong nonlinearity of the leading wave would contribute up to 0.52 m/s to its propagation speed. With a linear propagation speed of 2.69 m/s, the propagation speed of the leading wave is estimated to be 3.22 m/s, which is 0.38 m/s faster than the mean ISW case with an amplitude of 67 m.

### Energy in the ISW packet

Based on the moored temperature, salinity and velocity measurements, energy contained in the ISW packet was calculated (see Methods). At the trough of the leading wave, the maximum KE and APE densities were of 3.34 and 1.81 KJ/m^3^, respectively. The integrated KE and APE along the orientation of the ISW packet reached 3.84 and 4.29 GJ/m, respectively, which are two times of those of one prototypical ISW in the northern SCS reported by previous literature^15^. The energy of the baroclinic tides in the northern SCS, emanating from the LS that is known as the most energetic barotropic-to-baroclinic energy conversion region among the world oceans^24^, is considered to be the primary energy source for the enhanced vertical mixing and circulation in the deep SCS^25^,^26^. We should note that, the energy contained in this extreme ISW packet, which lasted only 3 hours, is comparable to the total energy in the diurnal and semidiurnal internal tides of a whole day.

### Measurements near Batan Island

**Figure 4:**
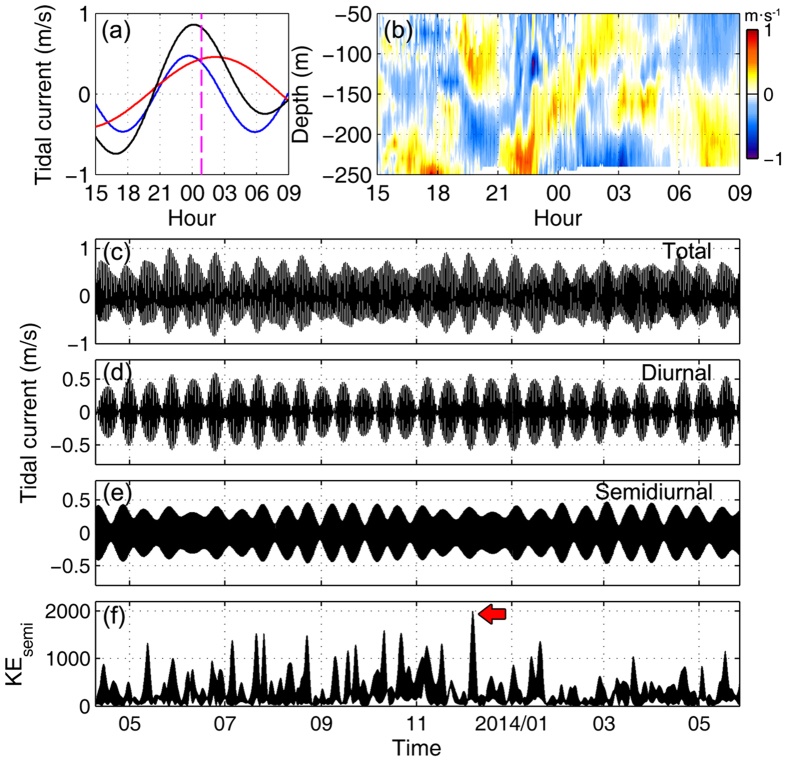
Mooring measurements at IW1 near Batan Island. **(a)** Time series of total barotropic tidal current (black line) around 00:00 4 December 2013 at IW1 and its semidiurnal (blue line) and diurnal (red line) components. The magenta dashed line denotes the estimated generation time of the extreme ISW. **(b)** Zonal baroclinic current between 50 and 250 m around 00:00 4 December 2013 at IW1. **(c–e)** Time series of total barotropic tidal current, diurnal tidal current and semidiurnal tidal current at IW1 from April 2013 to June 2014, respectively. **(f)** Depth-integrated KE density (J/m) of the semidiurnal internal tide. Figures are plotted using MATLAB R2013a (http://www.mathworks.com/).

It is generally accepted that the eastern ridge in the LS is the primary source region of ISWs^13^,^21^,^27^,^28^. To monitor the generation processes of ISWs, concurrent moored observations were conducted in the LS near Batan Island with the bottom depth of 329 m (mooring IW1 in Fig. 1b). By applying a band-pass filter to the time series of depth-averaged currents, the zonal semidiurnal (with periods of 9–15 h) and diurnal (with periods of 21–27 h) barotropic tidal currents at IW1 were obtained. Based on the stratification derived from the winter data of the WOA, we estimate that about 18.2 h is needed for ISW to propagate from IW1 to M10 according to the ratio between ISW propagation speed and linear phase speed^21^. Tracing back the extreme ISW signals at M10 to IW1, we find that the generation time of the extreme ISW well corresponded to the eastward total tidal current (marked by the magenta dashed line in Fig. 4a) over the generation site, consistent with previous studies^13^,^18^,^21^. No wave with such pattern as the extreme ISW at M10 was found in the baroclinic current measurements at IW1 (Fig. 4b). This result suggests that the generation mechanism of the extreme ISW might not be linked to the lee-wave generation mechanism.

From satellite remote sensing images, previous study demonstrated that ISWs in the northern SCS are developed from the internal tides through the mechanism of nonlinear steepening^19^. This point has been confirmed by follow-up field^18^,^21^,^29^ and numerical^28^,^30^ studies. Previous studies further suggested that the semidiurnal internal tide in the northern SCS is able to disintegrate into ISWs while the disintegration of diurnal internal tide is largely restrained due to the dispersion effect of the Earth’s rotation^29^,^31^,^32^. To examine the variation of the semidiurnal internal tide generation near Baton Island, a band-pass filter (with periods of 9–15 h) was applied to the time series of zonal baroclinic velocity measured at IW1. Fig. 4f shows that there existed a remarkable peak in the depth-integrated KE density of the semidiurnal internal tide in early-December, coinciding with the occurrence time of the extreme ISW at M10. Both semidiurnal and diurnal internal tides contribute to the formation of ISWs, but the former plays a dominant role, and therefore it is reasonable to attribute the occurrence of the extreme ISW at M10 to the powerful generation of semidiurnal internal tide in the LS.

## Discussion

**Figure 5:**
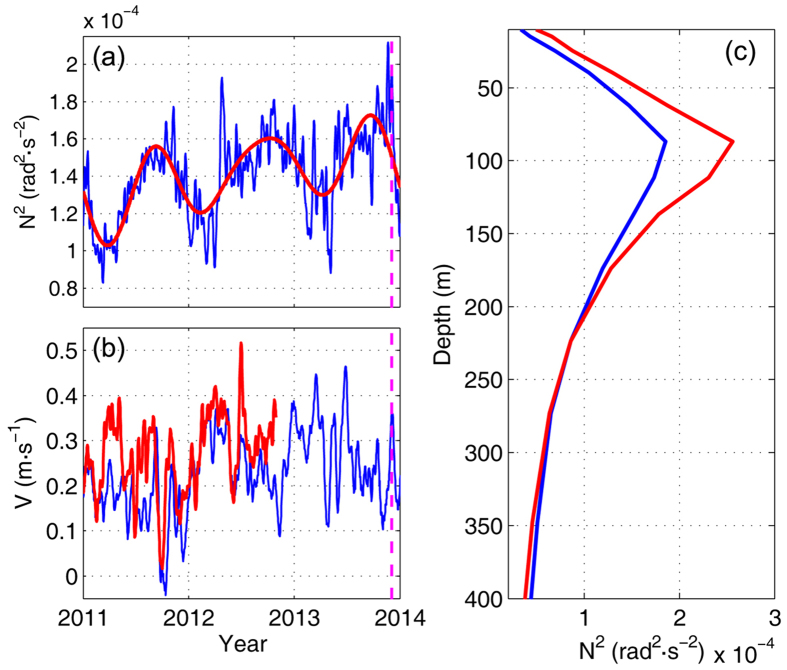
Results of the mooring measurements at KC1 and numerical simulation of HYCOM. **(a)** Time series of depth-averaged N^2^ (blue line) between 100 and 200 m around Batan Island calculated from the HYCOM product, and its 500-day low-passed result (red line). Magenta dashed line marks the occurrence time of the extreme ISW. **(b)** Time series of depth-averaged meridional velocities between 200 and 300 m from the ADCP measurements (red line) at KC1 and from the HYCOM product (blue line). **(c)** Spatially-averaged upper-layer stratification of the Kuroshio water (red line) along 18°N to the east of Luzon Island and the water around Batan Island (blue line). Figures are plotted using MATLAB R2013a (http://www.mathworks.com/).

Changes of barotropic tide and stratification are known as decisive factors that can affect the generation of baroclinic tides. By examining the changes of barotropic tide at IW1, we find that the barotropic tide around 4 December was not stronger than those during other spring-neap cycles (Fig. 4c–e). This result suggests that the variation of barotropic tide could not account for the generation of strong semidiurnal internal tide. To diagnose the variability of stratification over the source site of ISWs, we employ the hybrid coordinate ocean model (HYCOM) product (see Methods) to compensate for the lack of long-term observational temperature and salinity data in the LS. Based on the HYCOM salinity and temperature outputs, the depth-averaged buoyancy frequency squared N^2^ between 100 and 200 m around Batan Island (as marked by the black rectangle in Fig. 1b) was calculated. As shown in Fig. 5a, the value of N^2^ reached a peak around early-December in 2013, which coincided well with the occurrence of the extreme ISW. An eastward background flow over the generation site can amplify the generation of internal tides and the amplitude of ISWs^29^,^33^. In early-December of 2013, however, a westward background flow was present over the generation site of internal tides around IW1 (Supplementary Fig. S2), with a comparable magnitude to the average between March 2010 and June 2014. Hence, we think that the role of LS background flow in the formation of the extreme ISW was limited. Further, given that the observed barotropic tide at IW1 was not intensified, it is reasonable to consider that the increased stratification likely contributed to the formation of the extreme ISW in early-December of 2013.

It is noted that no ISW with a comparable amplitude as the extreme ISW was captured in 2011 and 2012 during the full-year measurements, indicating that there existed interannual variations of ISWs in the northern SCS. Superimposed on the seasonal cycle, Fig. 5a shows that the value of N^2^ exhibited a significantly increasing interannual trend from 2011 to 2013. In early-December of 2013, the intraseasonal variation of N^2^ further increased the stratification over the source site of ISWs. The superposition of the interannual trend and intraseasonal variation led to the peak of N^2^, the date of which matched the spring period of semidiurnal barotropic tide in early-December of 2013 (see Supplementary Fig. S3). We should note that large intraseasonal augments in stratification also occurred in 2011 and 2012 (e.g., April 2012). However, superimposed on a relatively lower level, the values of N^2^ during 2011 and 2012 were not comparable to that in early-December of 2013. This result suggests that the 3-year interannual increasing trend of N^2^ seemed to be a key basis for the generation of energetic internal tides and formation of the extreme ISW.

During the SIWE, mooring measurements over 25 months were collected in the eastern deep basin close to the LS (B3, from August 2010 to April 2011; M10, from April 2011 to April 2012, and from October 2013 to June 2014). In order to examine the long-term trend of ISWs, monthly-averaged maximum current velocity of ISWs were calculated, which show that the ISWs near the LS at B3 and M10 seemed to strengthen from late 2010 to early 2012, and reached a peak at the end of 2013 (Supplementary Fig. S4). Drawing an explicit conclusion that the ISWs continuingly became stronger from 2012 to 2013 requires the support of observations in 2013. However, considering that the barotropic tide in the LS varied little from year to year, we can safely assume that the strengthened ISWs from late 2010 to early 2012 near the LS at B3 and M10 were associated with the enhanced stratification near Batan Island.

Considering that the current and thermal structures in the LS are largely impacted by the Kuroshio^34^, it is necessary to examine whether the increasing trend of N^2^ from 2011 to 2013 was associated with the Kuroshio. As demonstrated in Fig. 5c, the averaged stratification of the Kuroshio along 18°N was obviously stronger than the water around Batan Island. From 2011 to 2012, mooring measurements^35^ showed that the Kuroshio velocity at its origin at mooring KC1 increased by as large as 0.15 m/s (red line in Fig. 5b), which would have carried more warm water downstream into the LS. The 200–300 m averaged HYCOM meridional velocity at KC1 also exhibited an increasing trend from 2011 to 2012 (blue line). The HYCOM-simulated Kuroshio continued to strengthen in the year 2013, though it decreased a little bit over the last five months. Considering that the Kuroshio water was more stratified than the LS water, we speculate that the increasing trend of N^2^ from 2011 to 2013 might be related to the strengthened Kuroshio.

Previous studies have shown that the Kuroshio at its origin strengthened during an La Niña event^36^,^37^. As we know that the La Niña event during 2011–2012 was one of the strongest on record, and the Niño 3.4 index continued to be negative in 2013. As the hotbed of highly stratified water, our analysis present here suggests that the variation of the Kuroshio might play the role of a bridge as the climatic-scale El Niño and Southern Oscillation (ENSO) event modulating weather-scale ISW event. Given the availability of the 4-year moored observations of ISWs, it is difficult to ascertain the response of ISWs in the northern SCS to the ENSO events. This, however, is an interesting topic that needs further study in future.

## Methods

### *In-situ* moored data

From 29 October 2013 to 9 June 2014, a subsurface mooring labeled as M10 was deployed in the deep water of the northern SCS, as part of the SIWE, with a water depth of 3847 m (20.57°N, 120.22°E). One upward-looking and one downward-looking Long Ranger 75-kHz ADCPs were mounted on the mooring at the depth around 560 m to collect the velocity data in the upper 1000 m. The sampling interval and vertical bin size of the ADCPs were set to 3 min and 16 m, respectively. Between 160 and 1060 m, a thermistor chain consisting of temperature loggers and CTDs was attached to the mooring to monitor both temperature and salinity. The sampling interval of temperature was 2 min. At the depths of 1565, 2175, 2782, 3289, and 3803 m, the current velocities were measured every 30 min by five RCMs. Five CTDs were installed just 5 m below the RCMs to record deep-ocean temperatures every 2 min.

At M10, a subsurface mooring was also deployed between 25 April 2011 and 5 April 2012. The mooring was equipped by one upward-looking 75-kHz ADCPs at 473-m depth, which monitored the velocity information in the upper 460 m every 5 min. Two RCMs and two CTDs were mounted on the mooring in the deep water between 3525 and 3730 m. The location, instrument setting and working time of this mooring are summarized in Supplementary Table S1.

In the eastern deep basin near M10, a subsurface mooring labeled as B3 (120.11°E, 20.71°N) was deployed between 12 August 2010 and 21 April 2011, at a water depth of 3745 m. Both upward and downward-looking ADCPs were mounted on the mooring at the depth of 511 m. Between 100 and 500 m, mooring B3 was equipped with a temperature chain. In the deep water, one RCM and one CTD were attached on the mooring at ~3520 m.

In the LS near Batan Island where the water depth is 329 m (20.55°N, 121.88°E), a subsurface mooring named IW1 was deployed from 8 April 2013 to 28 May 2014. One upward-looking Long Ranger 75-kHz ADCP was mounted at the depth of 279 m, measuring the velocity from 254 m to near the surface every 3 min with a 16-m vertical bin size.

To the east of Luzon Island at the origin of the Kuroshio, two moorings were successively deployed at the depth of ~3400 m from November 2010 to October 2012. Between 20 November 2010 and 10 July 2011, an upward-looking 75-kHz ADCP was mounted on the first mooring (18.02°N, 122.63°E) at the depth of ~700 m, measuring the velocity from ~200 to 700 m every 30 min. Between 11 July 2011 and 30 October 2012, both upward-looking and downward-looking ADCPs were mounted on the second mooring (17.96°N, 122.93°E) at the depth of ~500 m, which recorded the velocity information from ~1100 m to near the surface. In this study, the above two moorings are regarded at the same position (called KC1) because they were not far from each other. For more detailed descriptions of the two moorings, readers are referred to the published papers^35^,^38^.

Due to contamination by acoustic reflections near the air-sea interface, the velocity measurements of the ADCPs between the sea surface and 50-m depth were discarded. The vertical motions of the ADCPs were estimated by computing the time derivative of the pressure signals, and then they were subtracted from the vertical velocity records of the ADCPs to remove the influences of mooring tilt.

### HYCOM product

In addition to the *in-situ* moored data, we employ the HYCOM product, which assimilates satellite altimeter and sea surface temperature data together with available temperature and salinity profiles from XBTs, ARGO floats and moored buoys. The HYCOM product provides 1-day-averaged variables/fields with a fine spatial resolution (1/12° by 1/12°), which aims to resolve the circulation and eddy variability. In the vertical, the HYCOM temperature, salinity and velocities are interpolated to 33 levels at the depths of 0, 10, 20, 30, 50, 75, 100, 125, 150, 200, 250, 300, 400, 500, 600, 700, 800, 900, 1000, 1100, 1200, 1300, 1400, 1500, 1750, 2000, 2500, 3000, 3500, 4000, 4500, 5000, and 5500 m.

### Vertical mode analysis

The theoretical structure of vertical velocity *f*_n_ of mode-n internal wave can be obtained by numerically solving the Taylor-Goldstein equation:


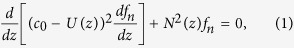


where 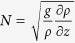 is the Brunt-Väisälä frequency and *c*_0_ is the linear phase speed. Here, the density profile *ρ*(z) is computed from the daily-averaged temperature and salinity profiles measured by mooring M10. The lack of stratification data near the surface and bottom was made up by the winter data of the WOA. The velocity data 30 min prior to the ISW arrival measured by the ADCPs and RCMs are used to construct the background current profile *U*(z). The background currents near the surface, where the velocity measurements of ADCP were contaminated by acoustic refractions, are complemented by assuming that the velocity was constant from 50-m depth to the surface. Below the deepest velocity measurement depth at 3803 m, the background current is assumed to linearly decrease to zero at the bottom.

The observed horizontal velocity *u* and isotherm displacement *η* can be expressed by the superposition of discrete modes 
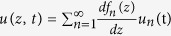
 and 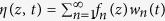, respectively. In this study, full-depth profiles of *u* and *η* at each time *t* are obtained by performing a least-squares inverse with the first five modes.

### The wave packet solution

The Kortevrieg–de Vries (KdV) equation


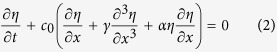


is generally used to describe characteristics of solitary waves. In equation (2) is the nonlinear parameter and *γ* is the dispersion parameter. In this study, these two parameters are calculated using the following formulas^31^:


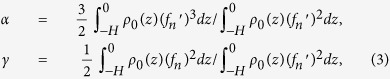


where the prime denotes the derivative of the variable with respect to depth. For a single soliton, the analytical solution to the KdV solution has a form of


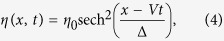


where *η*_0_ is the wave amplitude.


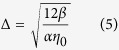


is characteristic width and


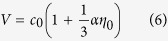


is wave propagation speed. However, due to the lack of information about trailing waves, the sech^2^ solution to the KdV equation is not suitable to analyze the characteristics of the multi-wave ISW packet.

To interpret the dynamics of the multi-wave ISW packet in Fig. 3c, the Dnoidal solution


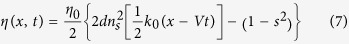


to the KdV equation is applied. Here, *k*_0_ is the wavenumber of the soliton at the packet rear, and *t* is the growth time of the packet. The Jacobi elliptic function dn_s_(X) of argument X is oscillatory quantity. The nonlinear parameter *s* is determined using the following equation,


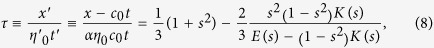


where *τ* is the space–time ratio, and *K*(s) and *E*(s) are complete elliptic integrals. The nonlinear parameter *s* decreases monotonically from unity at the leading edge to zero at the trailing edge, and the space-time ratio *τ* is verified to vary from 2/3 to −1. The Supplementary Fig. S5a schematically shows the Dnoidal solution. The leading wave with the nonlinear parameter *s* that equals to unity has a fully-nonlinear wave shape. When *τ* is larger than 2/3, the solution is assumed to be one half of the sech^2^ solution. In the Dnoidal solution, the nonlinear phase speed *V* is given by


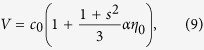


and the relationship of the short-wavelength wave number *k*_0_ to the parameters *η*_0_, *α* and *γ* is given by 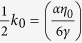. Moreover, the distance between successive solitary waves is given as


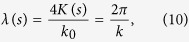


and therefore the time interval between two successive solitary waves (wave period) can be obtained by


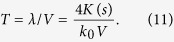


The multi-wave ISW packet in the ocean differs from the Dnoidal solution because its waveform gradually returns to the undisturbed depth after half of the semidiurnal tidal cycle. Assuming a propagation speed *c*_0_ of 2.5 m/s, the semidiurnal internal tides in the deep water of the northern SCS have wavelengths of approximately 100 km. Here, a recovery function


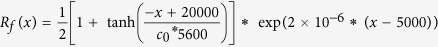


is adopted. As is shown in Supplementary Fig. S6, the trailing edge of the waveform returns to the starting position after the recovery.

## Calculation of the Energy in the Isw Packet

The measured time series of velocity and temperature are transformed to the space coordinate in the wave’s reference frame, 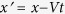. Then, the KE and APE of the ISW packet are computed through


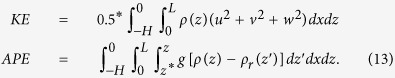


Here, *ρ*_r_(z) is the background density profile prior to the arrival of the ISW, and *z* ′ is the depth of the water particle in the background density profile. The velocity and stratification profile have been extrapolated to the surface and bottom using the mode analysis method.

## Additional Information

**How to cite this article**: Huang, X. *et al.* An extreme internal solitary wave event observed in the northern South China Sea. *Sci. Rep.* **6**, 30041; doi: 10.1038/srep30041 (2016).

## Supplementary Material

Supplementary Information
