# Supplementary material for: An extreme internal solitary wave event observed in the northern South China Sea
Source: Sci Rep. 2016 Jul 21;6:30041. doi: 10.1038/srep30041 (PMC4956752; doi:10.1038/srep30041)
Supplement: Supplementary Information [file srep30041-s1.doc]

**Supplementary Information**

An extreme internal solitary wave event observed in the northern South China Sea

Xiaodong Huang^1^, Zhaohui Chen^1,2^, Wei Zhao^1,2,*^, Zhiwei Zhang^1^, Chun Zhou^1^, Qingxuan Yang^1,2^, Jiwei Tian^1,2^

Physical Oceanography Laboratory/Qingdao Collaborative Innovation Center of Marine Science and Technology, Ocean University of China, 238 Songling Road, Qingdao 266100, P.R. China.

**^2^** Qingdao National Laboratory for Marine Science and Technology, 1 Wenhai Road, Qingdao 266200, P.R. China.

^*^**Corresponding author:** Wei Zhao (Physical Oceanography Laboratory/Qingdao Collaborative Innovation Center of Marine Science and Technology, Ocean University of China, 238 Songling Road, Qingdao 266100, P.R. China; Telephone: +86-0532-66786311; Email: weizhao@ouc.edu.cn)


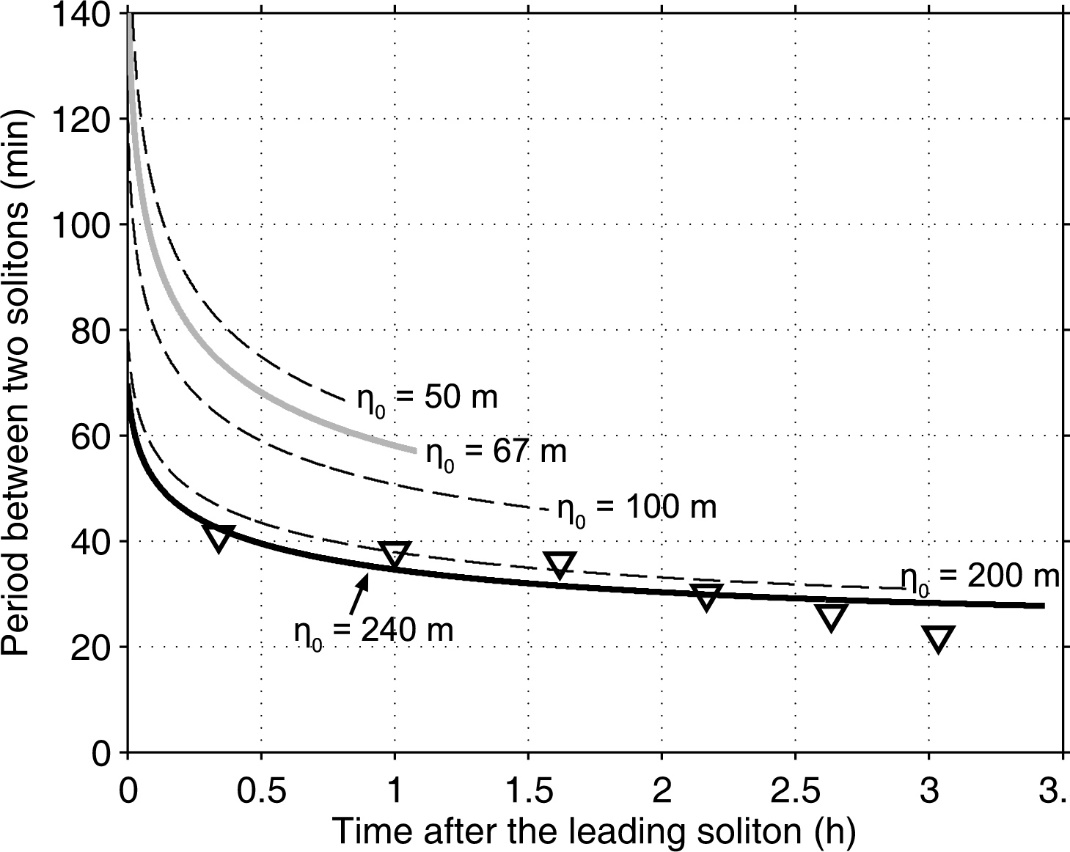


**Supplementary Figure S1 | Time intervals between two successive solitary waves in multi-wave ISW packets.** Triangles denote the observed time intervals in the extreme ISW packet. The various curves are the theoretical results predicted by the dnoidal solution with the same growth time *t* = 6.6 h under different *η*_0_.


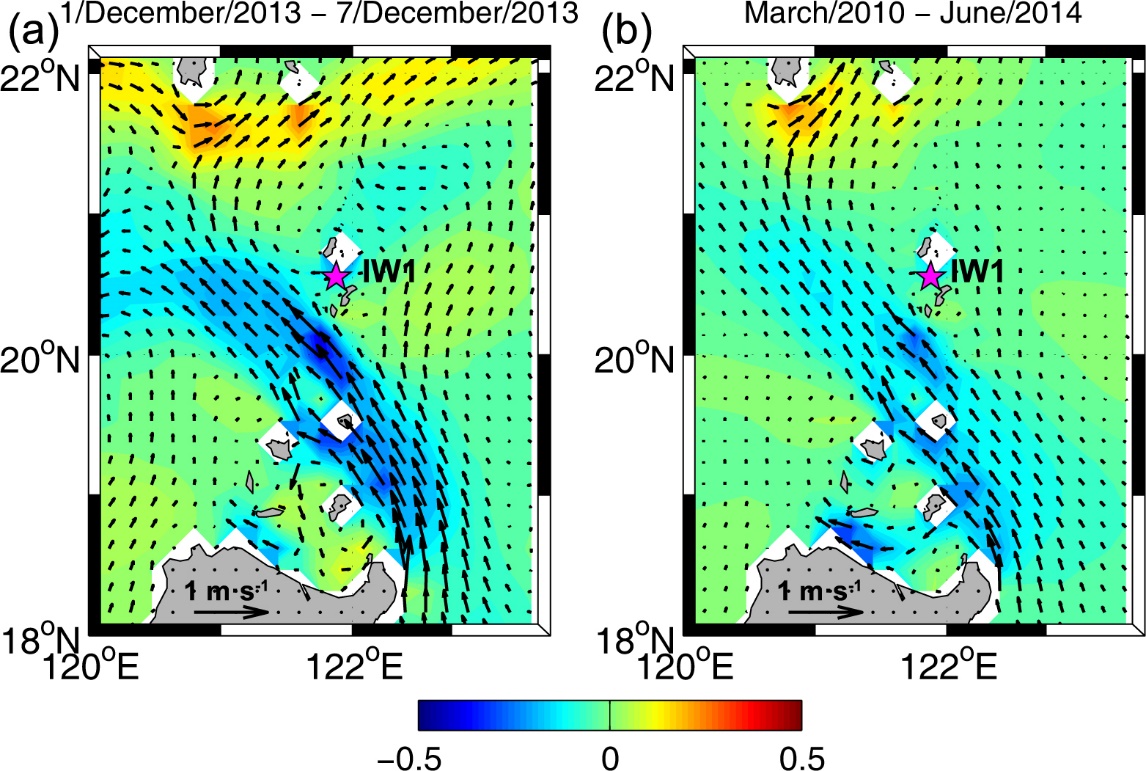


**Supplementary Figure S2 | Hydrological condition in the Luzon Strait.** **(a)** Temporally-averaged surface currents in early December of 2013, where the shadings indicate zonal surface currents (m/s). **(b)** The same as (a), except for the full experiment period between March 2010 and June 2014. Figures are plotted using MATLAB R2013a (http://www.mathworks.com/). The maps in this figure are generated by MATLAB R2013a with M_Map (a mapping package, http://www.eos.ubc.ca/~rich/map.html).


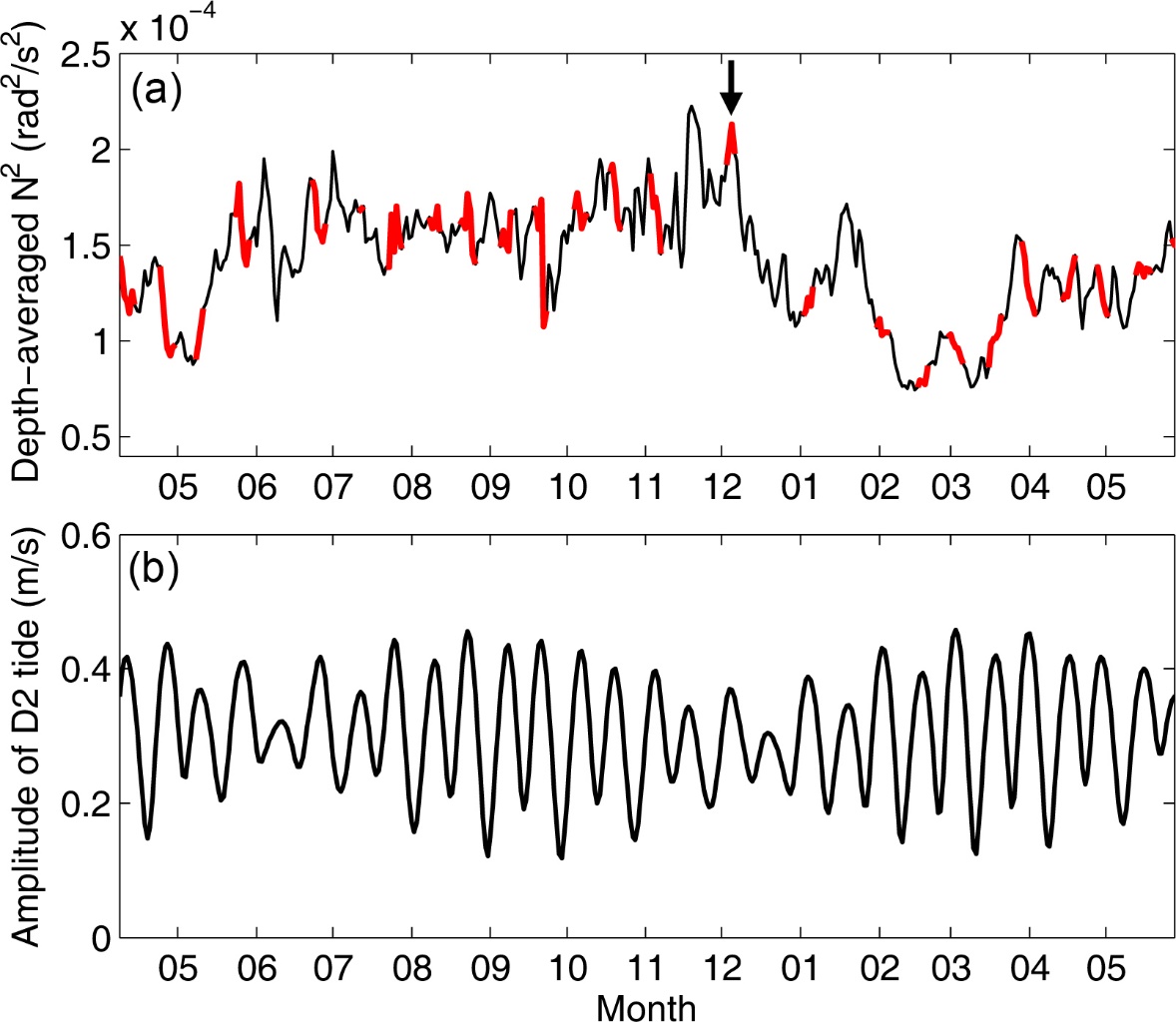


**Supplementary Figure S3 | Stratification and semidiurnal barotropic tidal current around Batan Island.** **(a)** Depth-averaged N^2^ between 100 and 200 m around Batan Island from 8 Apr 2013 to 28 May 2014. Bold red lines mark the periods when the amplitude of semidiurnal tidal current at IW1 was in excess of 0.35 m/s, and black arrow denotes the occurrence time of the extreme ISW event. **(b)** The measured amplitude of zonal semidiurnal barotropic tidal current at IW1 derived via the harmonic analysis with an overlapping window of 3 days using the time series of barotropic current.


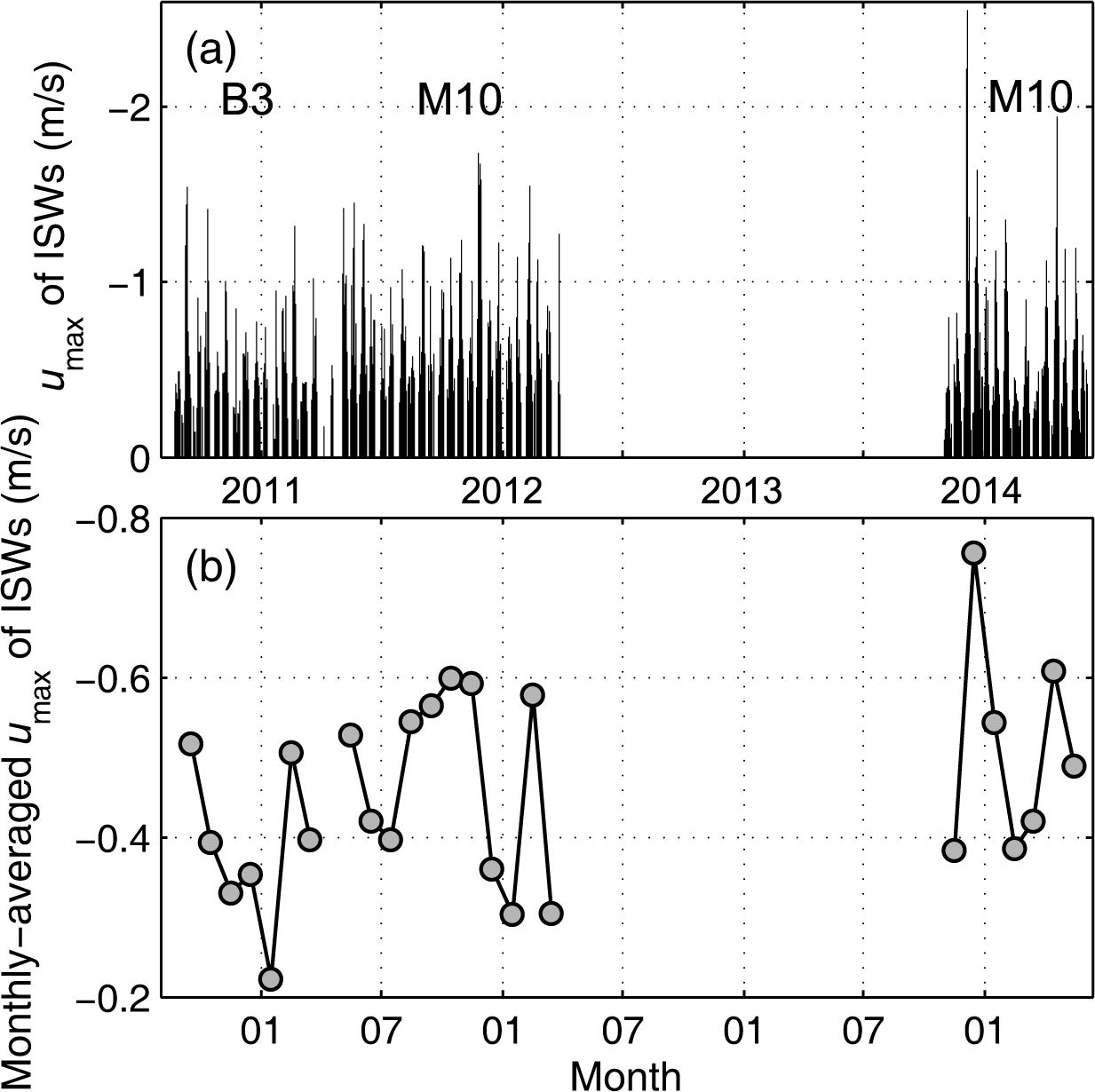


**Supplementary Figure S4 | ISWs measured in the eastern deep basin at B3 and M10. (a)** Occurrence time and maximum westward current velocity of ISWs measured at B3 and M10. **(b)** Monthly-averaged maximum westward current velocity of ISWs.


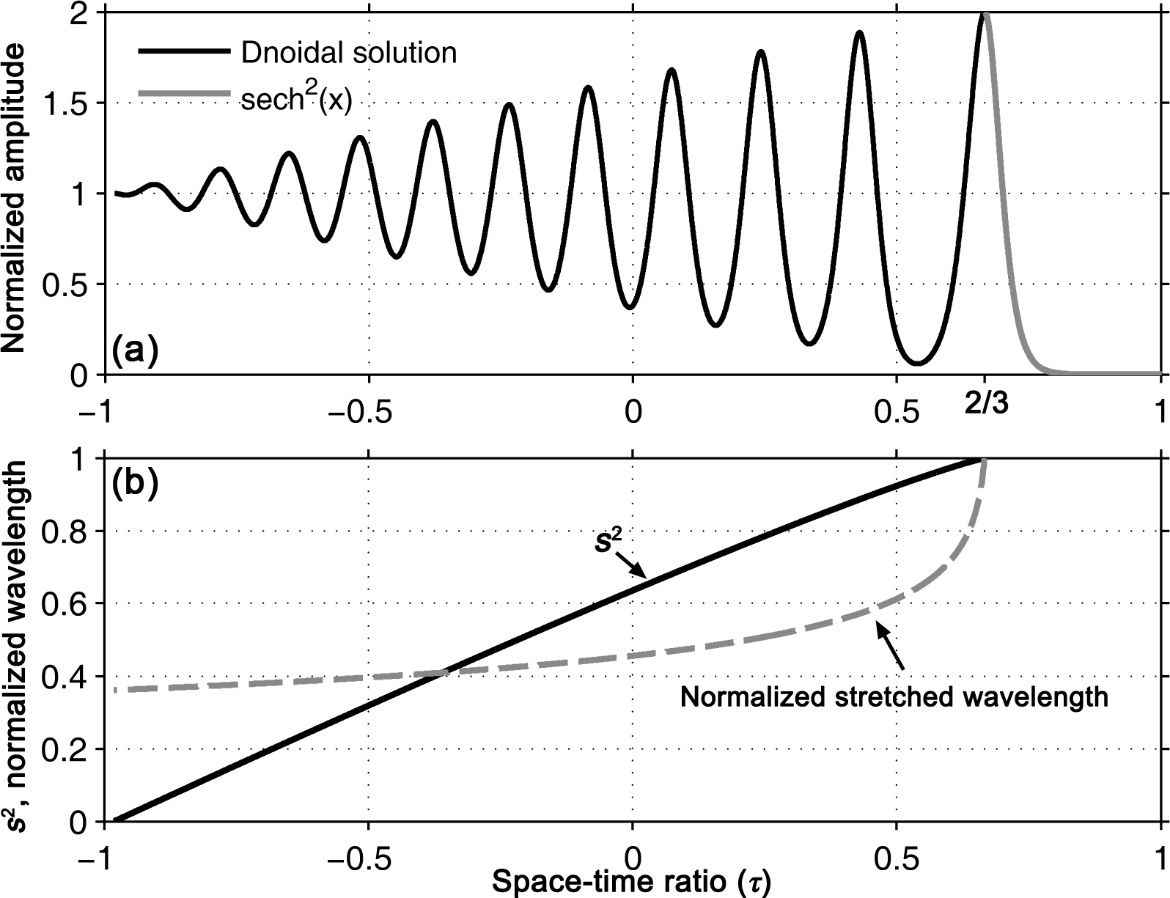


**Supplementary Figure S5 | Dnoidal solution to the KdV equation. (a)** The waveform with amplitude normalized by the leading wave. When *τ* is larger than 2/3, it is assumed to be one half of a sech^2^(x) soliton. **(b)** Squared nonlinear parameter and normalized stretched wavelength.


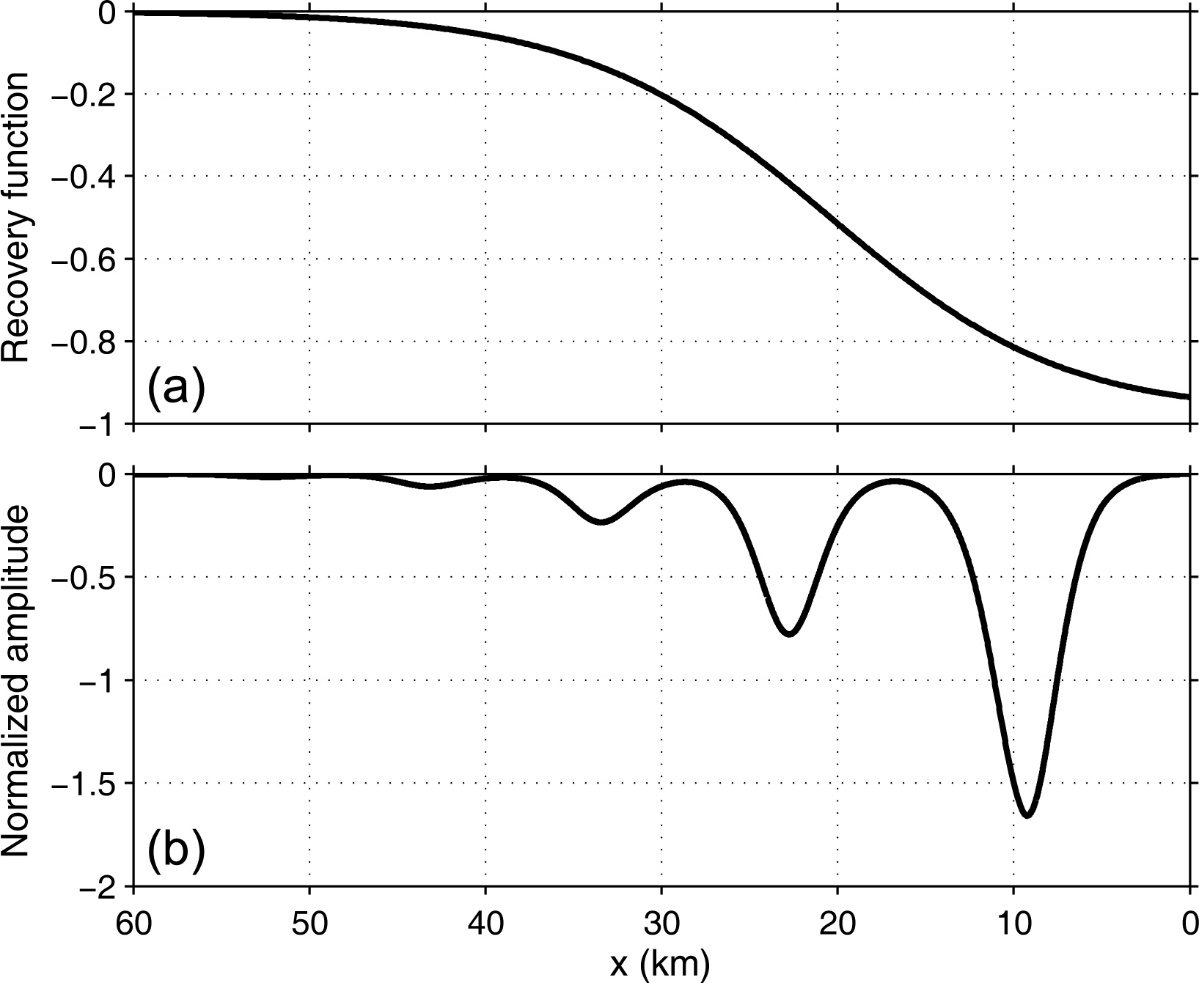


**Supplementary Figure S6 | Recovery of trailing waves in the dnoidal solution.** **(a)** The recovery function used in this study. **(b)** The waveform of dnoidal solution in Supplementary Fig. S5a after being recovered.

**Supplementary Table S1 | Location of Moorings and Detailed Settings of Instruments**

| Mooring | Observation Period | Longitude, Latitude | Water Depth (m) | Instrument | Instrument Depth (m) | Range Depth (m) | Bin Size (m) | Sample Interval (min) |
| --- | --- | --- | --- | --- | --- | --- | --- | --- |
| M10 | Oct/29/2013– Jun/09/2014 | 120.22°E, 20.57°N | 3847 | Temperature chains | ** | 160-180  180-560  560-760  760-1060 | 10  20  50  100 | 2 |
|  |  |  |  | 75 kHz ADCP (up)  75 kHz ADCP (dw) | 560  560 | 58-538  578-994 | 16 | 3 |
|  |  |  |  | RCMs | 1565, 2175, 2782, 3289, 3803 | ** | ** | 30 |
|  |  |  |  | CTDs | 160, 200, 260, 560, 1060, 1570, 2180, 2787, 3294, 3808 | ** | ** | 2 |
|  | Apr/25/2011 - Apr/05/2012 | 120.20°E, 20.54°N | 3831 | 75 kHz ADCP (up) | 473 | 28-460 | 8 | 5 |
|  |  |  |  | RCMs | 3525,3725 | ** | ** | 60 |
|  |  |  |  | CTDs | 3530,3730 | ** | ** | 30 |
| B3 | Aug/12/2010 - Apr/21/2011 | 120.11°E, 20.71°N | 3745 | Temperature chains | ** | 100-500 | ** | 3 |
|  |  |  |  | 75 kHz ADCP (up)  75 kHz ADCP (dw) | 511  511 | 0-494  528-1028 | 8 | 5  60 |
|  |  |  |  | RCM | 3520 | ** | ** | 60 |
|  |  |  |  | CTDs | 100, 200, 300, 3525 | ** | ** | 3 |
| IW1 | Apr/08/2013  -  May/28/2014 | 121.88°E,  20.55°N | 329 | 75 kHz ADCP (up) | 279 | 0-254 | 16 | 3 |
